# Benign emptying of the post-pneumonectomy space: A case report

**DOI:** 10.1016/j.ijscr.2021.105699

**Published:** 2021-02-24

**Authors:** Nozomu Motono, Masahito Ishikawa, Shun Iwai, Yoshihito Iijima, Katsuo Usuda, Hidetaka Uramoto

**Affiliations:** Department of Thoracic Surgery, Kanazawa Medical University, 1-1 Daigaku, Uchinada, Ishikawa, Japan

**Keywords:** BEPS, benign emptying of the post-pneumonectomy space, BPF, bronchopleural fistula, CT, computed tomography, EPP, extrapleural pneumonectomy, Bronchopleural fistula, Benign emptying of the post-pneumonectomy space, Non-small cell lung cancer

## Abstract

**Introduction and importance:**

Benign emptying of the post-pneumonectomy space (BEPS) is characterized by a decreased air-fluid level after pneumonectomy, albeit without the presence of bronchopleural fistula (BPF).

**Case presentation:**

A 66-year-old man with lung cancer diagnosed clinical stage IIIA received right pneumonectomy. The air-fluid level of the right pleural cavity suddenly dropped on the 19th postoperative day. Although we were unable to detect the fistula of the bronchial stump using bronchoscopy, we performed re-operationt. Although a pressure of 30 cmH_2_O was applied into the airway and air leakage from the bronchial stump was not detected, we covered the bronchial stump with the omentum. Because the air-fluid level of the right pleural cavity decreased 9 days after the 2nd operation without any symptom, we diagnosed the condition as BEPS.

**Clinical discussion:**

If a fistula of the bronchial stump cannot be identified using bronchoscopy or thoracoscopy, then determining whether it is BEPS or microscopic BPF can be difficult.

**Conclusion:**

It is currently unclear what the optimal strategy is in this case.

## Highlights

• Bronchopleural fistula (BPF) is recognized by a decrease in the air-fluid level within the pleural cavity as observed during chest radiology.
• Benign emptying of the post-pneumonectomy space (BEPS) is also characterized by a decreased air-fluid level after pneumonectomy, albeit without the presence of BPF.
• If a fistula of the bronchial stump cannot be identified, determining whether it is BEPS or microscopic BPF can be difficult.

## 1 Introduction

The incidence of bronchopleural fistulae (BPF) following pneumonectomy has been reported to be 0.8% [1]. However, decreases in air-fluid levels following pneumonectomy can still occur in the absence of BPF, particularly in the case of benign emptying of the post-pneumonectomy space (BEPS) [2]. Herein, we describe a case in which a decrease in air-fluid level occurred after pneumonectomy without the presence of detectable BPF. This case report has been reported in line with SCARE Criteria [3].

## 2 Case report

**Fig. 1:**
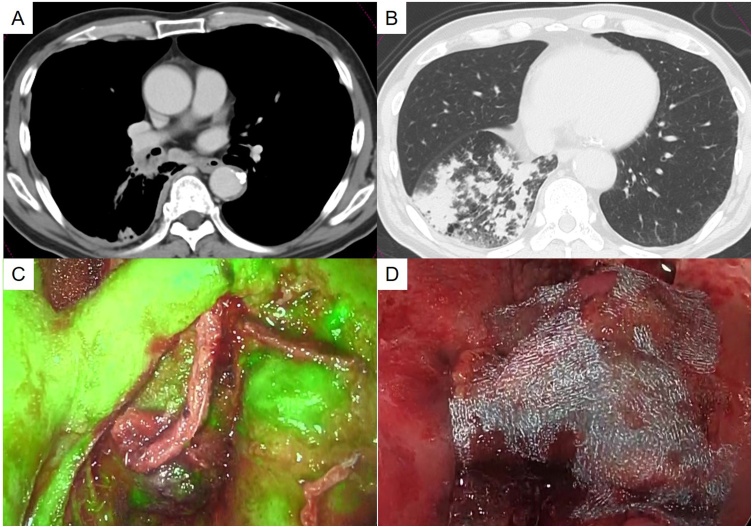
(A) Chest computed tomography revealed a mass lesion in the truncus intermedius of the right lung and swelling of the inferior tracheobronchial lymph nodes. (B) Pulmonary infiltration shadows were detected in the right lower lobe using computed tomography. (C) The blood flow of the bronchial stump was assessed using an ICG injection under fluorescence navigation. (D) The bronchial stump was covered by a stemless pericardial fat pad.

During medical examination, we detected an abnormal shadow in the chest of a 66-year-old man who was admitted to our hospital. Chest computed tomography (CT) revealed a mass lesion in the truncus intermedius of the right lung and swelling of the inferior tracheobronchial lymph nodes (Fig. 1A). Furthermore, CT scanning showed pulmonary infiltration shadows of the right lower lobe (Fig. 1B). Based on the findings of transbronchial lung biopsy, we gave the diagnosis of squamous cell carcinoma. Although we diagnosed the lung cancer as clinical stage IIIA (cT1cN2M0), we decided that chemoradiotherapy should not be performed due to the presence of obstructive pneumonia in the right lower lobe occurred by lung cancer. Instead, we planned to perform primary surgery.

We performed the operation via an anterolateral incision made with the patient in the lateral position. Although we did try to perform right middle and lower sleeve lobectomy, right pneumonectomy was ultimately chosen since the cancer had already spread to the right upper bronchus. We assessed the blood flow in the bronchial stump using an ICG injection under fluorescence navigation (VISERA ELITE II; Olympus, Tokyo, Japan) (Fig. 1C), which we then covered with a stemless pericardial fat pad (Fig. 1D).

**Fig. 2:**
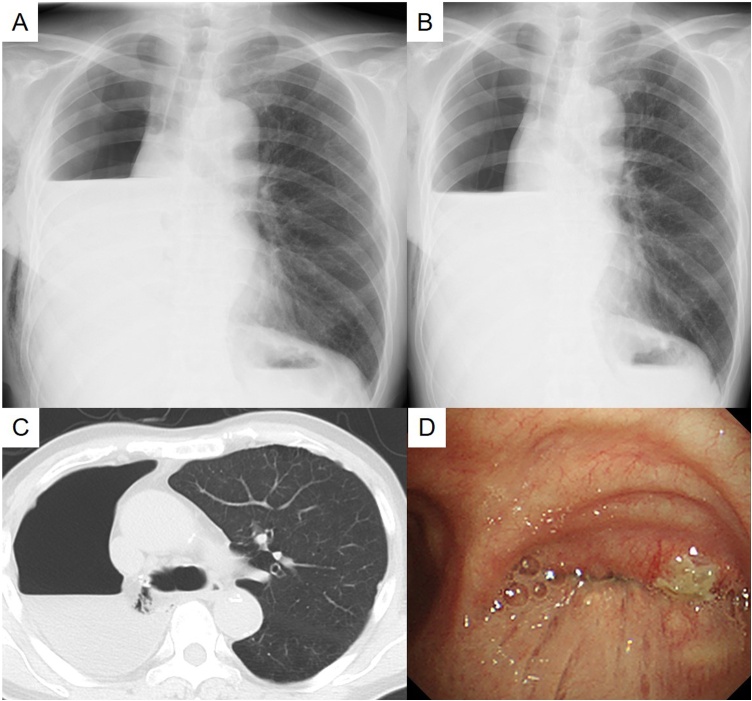
(A) Chest radiogram taken on the 4th postoperative day. (B) The air-fluid level of the right pleural cavity suddenly decreased on the 19th postoperative day. (C) The air was then detected around the bronchial stump using computed tomography. (D) Bronchoscopy was not able to detect the fistula of the bronchial stump.

**Fig. 3:**
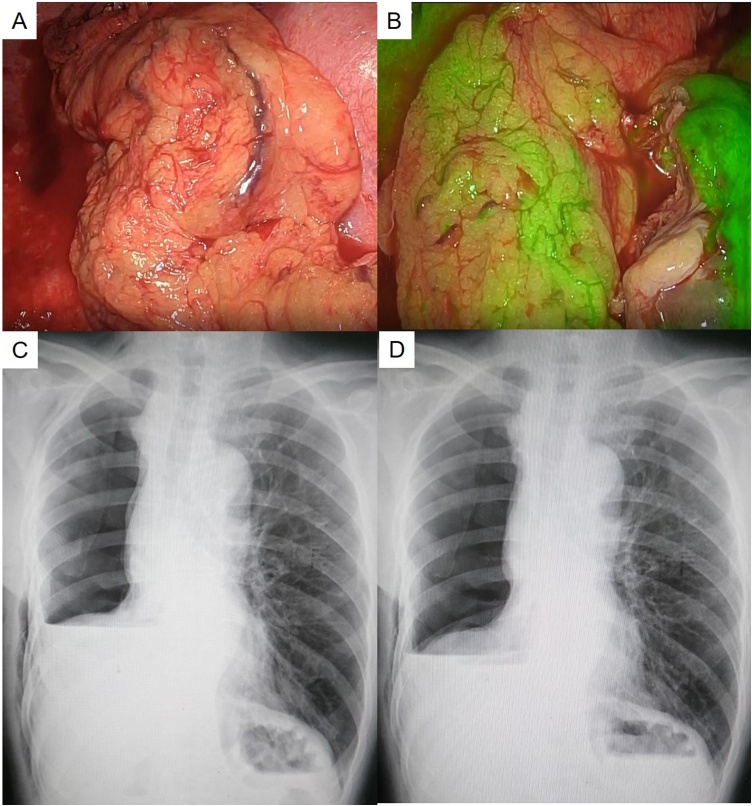
(A) The bronchial stump was covered by the omentum. (B) The blood flow in the omentum was assessed using an ICG injection under fluorescence navigation. (C) The air-fluid level of the right pleural cavity decreased after 9 post-2nd operative days. (D) The air-fluid level of the right pleural cavity had declined further 14 days after the 2nd operation.

The air-fluid level of the right pleural cavity suddenly dropped on the 19th postoperative day (Fig. 2A, B), and the air was detected around the bronchial stump using chest CT (Fig. 2C). Although we were unable to detect the fistula of the bronchial stump using bronchoscopy (Fig. 2D), we performed re-operation, as microscopic BPF could not be ruled out. Although a pressure of 30 cmH_2_O was applied into the airway, air leakage from the bronchial stump was not detected. However, we covered the bronchial stump with the omentum (Fig. 3A). The blood flow in the omentum was assessed using an ICG injection under fluorescence navigation (Fig. 3B).

The air-fluid level of the right pleural cavity decreased 9 days after the 2nd operation (Fig. 3C). Since the air-fluid level of the right pleural cavity declined further on the 14th day after the 2nd operation (Fig. 3D), we diagnosed the condition as BEPS. The patient was discharged on postoperative day 18 without any symptoms.

## 3 Discussion

BEPS has been reported to occur in 0.29–0.65% of pneumonectomies’ [2,4]. Three escape routes have been postulated with regard to pleural fluid: (1) congenital diaphragm fenestration; (2) a defect in the diaphragm and peritoneum created during the extrapleural pneumonectomy (EPP); and (3) loose chest wall closure [2]. In our case, we did not detect diaphragm fenestration in the 2nd operation, we did not perform EPP, and we found no escaped pleural fluid in the soft tissue of the chest wall. Another possible explanation of asymptomatic decreases in air-fluid levels within the pleural cavity is the so-called “occult bronchopleural fistula” [2]. It has been postulated that the pleural fluid cannot pass through the air and, as a result, a large inoculum of bacteria with the potential to cause intrapleural infection does not enter the pleural space due to the small size of the fistula. Instead, the air enters into the pleural cavity only during forceful coughing.

Although BPF is often necessary in BEPS patients due to the potentially fatal risks of empyema and aspiration pneumonia associated with the condition [5,6], the optimal management strategy remains unclear. Strict clinical criteria for BEPS have been reported, and the diagnosis can be given without any signs of fluid expectoration, fever, elevated serum white blood cell counts, fistula of the bronchial stump during bronchoscopy, or pleural fluid culture [2]. Close clinical observation, such as performing a chest radiogram every 1–2 weeks until the pleural fluid level replenishes to the level of the bronchial stump or beyond, has been recommended for BEPS diagnosed using the strict clinical criteria in cases without surgical intervention. In our case, the strict clinical criteria for BEPS were met after both the 1st and 2nd operations, and the clinical course after the latter was stable without surgical intervention. Close clinical observation of BEPS diagnosed according to the strict clinical criteria appears to be warranted, as it may prevent unnecessary surgical intervention.

## 4 Conclusion

If a fistula of the bronchial stump cannot be identified using bronchoscopy or thoracoscopy, then determining whether it is BEPS or microscopic BPF can be difficult. It is currently unclear what the optimal strategy is in this case.

## Declaration of Competing Interest

The authors report no declarations of interest.

## Funding

There are no external funding resources for the study.

## Ethical approval

Ethical approval not required.

## Consent

Written informed consent was obtained from the patient for publication of this case report and accompanying images. A copy of the written consent is available for review by the Editor-in Chief of this journal on request.

## Author contribution

Nozomu Motono performed the research, collected and analyzed the data and wrote the paper. Masahito Ishikawa, Shun Iwai, Yoshihito Iijima, and Katsuo Usuda contributed to sample collection. Hidetaka Uramoto contributed to supervision of this study and revision of the manuscript.

## Registration of research studies

Not Applicable.

## Guarantor

Hidetaka Uramoto.

## Provenance and peer review

Not commissioned, externally peer-reviewed.

## References

[1] Shapiro M., Swanson S.J., Wright C.D. (2010). Predictors of major morbidity and mortality after pneumonectomy utilizing in the Society for Thoracic Surgeons General Thoracic Surgery Database. Ann. Thorac. Surg..

[2] Merritt R.E., Reznik S.I., DaSilve M.C. (2011). Benign emptying of the postpneumonectomy space. Ann. Thorac. Surg..

[3] Agha R.A., Franchi T., Sohrabi C., Mathew G., for the SCARE Group (2020). The SCARE 2020 guideline: updating consensus surgical CAse Report (SCARE) guidelines. Int. J. Surg..

[4] Kanakis M.A., Misthos P.A., Tsimpinos M.D. (2015). Benign emptying of the post-pneumonectomy space: recognizing this rare complication retrospectively. Interact. Cardiovasc. Thorac. Surg..

[5] Lois M., Noppen M. (2005). Bronchopleural fistulas. An overview of the problem with special focus on endoscopic management. Chest.

[6] Puskas J.D., Mathisen D.J., Grillo H.C. (1995). Treatment strategies for bronchopleural fistula. J. Thorac. Cardiovasc. Surg..

